# Adolescent pregnancy in the time of COVID-19: what are the implications for sexual and reproductive health and rights globally?

**DOI:** 10.1186/s12978-022-01505-8

**Published:** 2022-11-05

**Authors:** Sylvester Reuben Okeke, Dina Idriss-Wheeler, Sanni Yaya

**Affiliations:** 1 Centre for Social Research in Health, UNSW Sydney, Sydney, Australia; 2 Interdisciplinary School of Health Sciences, University of Ottawa, Ottawa, Canada; 3 School of International Development and Global Studies, Faculty of Social Sciences, University of Ottawa, 120 University Private, Ottawa, ON K1N 6N5 Canada; 4 The George Institute for Global Health, Imperial College London, London, UK

## Abstract

The COVID-19 pandemic has aggravated pre-existing challenges associated with adolescents’ sexual and reproductive health and rights (SRHR). Evolving evidence suggest that it could adversely impact the progress made towards improving sexual and reproductive health outcomes among young people. The pandemic has stalled achievements in reducing adolescent pregnancy and child marriage by reinforcing contextual and structural determinants of these reproductive health outcomes, especially among girls. The pandemic has increased disruptions to schooling, decreased access to sexual and reproductive health services and compounded pre-existing socio-economic vulnerabilities. The consequences of neglecting adolescent sexual and reproductive health services over the past 2 years, to focus on COVID-19, continue to emerge. This commentary argues for targeted and responsive approaches to adolescent SRHR that tackle preventable consequences resulting from inequities faced by adolescents globally, particularly girls.

## Introduction

Adolescents’ sexual and reproductive health and rights (SRHR) are continuously undermined; health services and access are lacking, and little attention is paid to equipping adolescents with the autonomy, empowerment and education to make decisions regarding their SRHR [1]. In this commentary, we discuss how the global COVID-19 pandemic has exacerbated existing issues and hindered progress towards adolescent SRHR outcomes (i.e., child marriage, access to safe abortions/contraception, access to education, maternal health, sexual violence). Unfortunately, adolescent girls bear the brunt of this social injustice, leading to increased negative health and socioeconomic outcomes.

## Adolescent pregnancy and sexual and reproductive health and rights

Before the outbreak of the COVID-19 pandemic, adolescent pregnancy was already a major public health concern globally, with developing countries disproportionately affected [2–5]. Adolescent pregnancy within or outside the contexts of (child) marriage poses a significant health and developmental challenge [5, 6]. Unplanned pregnancy among adolescent girls occurring outside the context of marriage, reduces likelihood of completing education and may also lead to forced marriage. According to UNICEF, millions of girls worldwide are married before their 18th birthday [6]. When girls are married off early, they are robbed of their childhood, denied education, and deprived of the prospects of fully developing their potential [2, 6]. Additionally, adolescent pregnancy has been associated with health and socioeconomic consequences [7].

Adolescent girls are less likely to be psychologically and physiologically ready for pregnancy, childbirth and childcare [6–8]. Health-related evidence shows that complications resulting from pregnancy and childbirth are the leading cause of death for girls aged 15–19 years, globally [9, 10]. Also, it is estimated that 3.9 of the 5.6 million abortions that occur among adolescent girls aged 15–19 years annually, are unsafe, thereby contributing to maternal mortality, morbidity and lifelong reproductive health problems [11]. Moreover, babies born to adolescent mothers have higher risks of low birth weight, preterm delivery and severe neonatal conditions [12–14].

Socio-economically, adolescent pregnancy is more likely to result in child or forced marriage. It can lead to adolescent girls dropping out of school, thereby impacting their future education and job opportunities [2, 6, 15]. In addition, adolescent pregnancy occurring outside the context of marriage may have social consequences such as stigma, rejection and/or violence by partner, parents or peers [7, 8, 16]. Consequently, the impact of adolescent pregnancy––within or outside marriages––is not restricted to adolescent girls and their families, but extends to both societal and generational spheres.

Projections before the outbreak of the COVID-19 pandemic indicate that 100 million girls will become child brides over the next decade [6]. This projection is a far cry from the global goal of ending child marriage by 2030 [6, 17]. Concerningly, this projection has been compounded by the COVID-19 pandemic. It is estimated that over the next decade, ten million girls will be at risk of becoming child brides because of the impact of the COVID-19 pandemic [6]. Consequently, the pandemic threatens the progress that has been made over the last decade in averting 25 million child marriages by reducing prevalence from 1 in 4 to 1 in 5 girls [6].

## COVID-19 pandemic and adolescent pregnancy

Disruptions associated with the COVID-19 pandemic reinforced pre-pandemic vulnerabilities to adolescent pregnancy and child marriage. A recent review of evidence on adolescent pregnancy in sub-Saharan Africa showed that the pandemic aggravated pre-existing determinants of adolescent pregnancy [18]. One common determinant of adolescent pregnancy is access to education [2–4, 19]. Evidence across the world indicates that school closures [6, 20], socio-economic distress [19, 21], disruptions to sexual and reproductive health (SRH) services [6] and increased sexual violence [7, 21] may have contributed to adolescent pregnancy and child marriages during the COVID-19 era. Lockdown was a major strategy and policy response to the COVID-19 outbreak. In the early days of the outbreak, and when no vaccine was available, total and protracted lockdowns resulted in the closure of schools worldwide. While some countries, depending on their socio-economic and infrastructural development, transitioned to online learning, there was total shutdown of schooling in most of the less developed countries. Staying out of school increased the vulnerabilities of adolescent girls in debuting sex or increasing sexual activity. Notably, this increase was occurring within the context of disrupted access to contraceptives and other SRH services.

A Kenyan-based study [21]––which compared COVID-19 and pre-COVID-19 cohorts—showed that more adolescents dropped out of school (9.7% vs. 3.0%), debuted sex (47.4% vs. 25.5%) and reported a pregnancy incident (10.9% vs. 5.2%). In addition, a systematic review of studies that examined the impact of the COVID-19 pandemic on adolescents’ SRH in low- and middle-income countries, linked school closure to increased rates of early marriages [22]. Similarly, COVID-19 related disruptions to schooling, and the economic hardship brought about by the pandemic, have been linked to early marriage among Indonesian adolescents aged 14–17 years [20]. Moreover, closure of schools, due to the COVID-19 pandemic could impact school-delivered reproductive and sexual health interventions [23]. Beyond pregnancy and early marriage, the COVID-19 pandemic also compounded chronic stress associated with teen parenting [24]. Even before the pandemic, adolescent parents were more likely to report feeling overwhelmed with the demand of childcare and parenting, partly due to economic instability and inadequate access to health care [25].

Furthermore, COVID-19-related disruptions to adolescent sexual and reproductive health services—which were less effective or even non-existent, especially in low- and middle-income countries (LMICs) [7, 26, 27]—may have also contributed to increased adolescent pregnancy. The pandemic compounded knowledge gaps, misconceptions and access to condoms and other contraceptives among young people [28–31]. Evidence suggests that sexual activity among adolescents and young adults co-existed with disruptions to SRH services during COVID-19 [28, 31]. In fact, fewer adolescents reported condom (47.8% vs. 52.2%) and hormonal contraceptive (4.1% vs. 7.0%) use after the pandemic compared to before [21].

Of note, priority shift to fighting the COVID-19 pandemic may have also contributed to poorer access to adolescent SRH information and services, thereby elevating risks for poor sexual and reproductive health outcomes [6, 28, 31]. In addition, the fear of contracting SARS-CoV-2 at hospitals may have also contributed to reduced seeking of and access to contraceptives. This is supported by evidence from the Ebola Virus Disease epidemic in some West African countries which showed the use of condoms and other contraceptives plummeted by a range of 90–95% [4]. This sharp drop in contraceptive access resulted in increased number of unplanned pregnancies among women and adolescent girls.

Moreover, the likelihood of sexual violence in increasing adolescent pregnancy during the COVID-19 lockdowns, cannot be ignored. Before the pandemic, evidence suggested that sexual violence contributes significantly to adolescent pregnancy [7]. Sexual violence against girls and women is commonly reported with pre-COVID-19 estimates of more than one third of girls experiencing coerced sexual debut in some countries [16]. COVID-19 restrictive lockdowns resulted in closure of schools, workplaces and settings where ‘fun-seekers’ may get sexual gratification. Spending more time in lockdown also implies that adolescent girls may experience greater exposure to perpetrators of sexual violence, which may include family members and other known people [4, 32, 33]. Compared to pre-COVID cohorts, a greater proportion of adolescent girls in Kenya during the pandemic (3.2% vs. 1.4%) reported experiencing sexual violence. Disruptions to condom and contraceptives services during COVID-19 amplified consequences of sexual violence by not only increasing the risk of pregnancy, but also of sexually transmissible blood-borne viruses (e.g., HIV) and/or infections (e.g., chlamydia).

## Promoting adolescent sexual and reproductive health and rights

The COVID-19 pandemic is not the first, neither will it be the last pandemic or public health emergency that the world will experience. The Monkeypox has recently been declared as a public health emergency of international concern [34]. Ample evidence has shown that adolescent pregnancy increases in times of public health emergencies as evidenced by the Ebola Virus Disease epidemic and the near-endemic COVID-19 state. Thus, beyond narratives, a key concern should be on how to leverage the experiences drawn from these public health emergencies to promote, reinforce and enable adolescent SRHR. A recent SRH profile of 132 countries highlighted the need for massive investment in sexual and reproductive health care, especially in LMICs [26].

Against this backdrop, it is important to establish a strong system for adolescent and young adult sexual and reproductive health services. These services need to be agile and robust to remain responsive even in the face of a public health emergency. While it is basic human instinct to stratify healthcare needs and prioritize efforts during a public health emergency, such prioritization must not neglect other health problems which might be *tips of larger icebergs*. Consequences of neglecting SRH services to focus on COVID-19 in the past two years continue to emerge, and the resulting lessons learned must go beyond documenting what happened. Instead, these lessons must spur action to fast-track global responses to addressing the SRH needs of adolescents.

## Conclusion

The WHO states that “the right to health…includes freedoms and entitlements…freedoms to control one’s health and body and to be free from interference…and entitlements … the right to a system of health protection that gives everyone an equal opportunity to enjoy the highest attainable level of health” [35]. With this pandemic and previous epidemics, negative adolescent SRHR outcomes are not surprising. Yet, we continue to be caught unprepared and unresponsive during emergencies, falling short to meet this basic human rights standard to health. We are failing to provide our adolescents, particularly girls, with the proper SRH services, policies, opportunities, and autonomy to access care, make decisions and live healthy and productive lives to their full potential. There needs to be expanded access to SRH services and education––beyond just information––by ensuring individual, interpersonal and structural level barriers are addressed to reach this at-risk population. This is especially true in LMICs, where the COVID-19 pandemic escalated the problem of poor access to SRH services.

## Data Availability

Not applicable.
